# CD8^+^ T cells in brain injury and neurodegeneration

**DOI:** 10.3389/fncel.2023.1281763

**Published:** 2023-11-21

**Authors:** Zhaolong Zhang, Zhongying Duan, Yu Cui

**Affiliations:** ^1^Department of Interventional Radiology, The Affiliated Hospital of Qingdao University, Qingdao, Shandong, China; ^2^Institute of Neuroregeneration and Neurorehabilitation, Qingdao University, Qingdao, Shandong, China; ^3^Qingdao Medical College, Qingdao University, Qingdao, China

**Keywords:** CD8^+^ T cells, brain injury, neurodegeneration, ischemic stroke, traumatic brain injury, Alzheimer’s disease, Parkinson’s disease

## Abstract

The interaction between the peripheral immune system and the brain is increasingly being recognized as an important layer of neuroimmune regulation and plays vital roles in brain homeostasis as well as neurological disorders. As an important population of T-cell lymphocytes, the roles of CD8^+^ T cells in infectious diseases and tumor immunity have been well established. Recently, increasing number of complex functions of CD8^+^ T cells in brain disorders have been revealed. However, an advanced summary and discussion of the functions and mechanisms of CD8^+^ T cells in brain injury and neurodegeneration are still lacking. Here, we described the differentiation and function of CD8^+^ T cells, reviewed the involvement of CD8^+^ T cells in the regulation of brain injury including stroke and traumatic brain injury and neurodegenerative diseases, such as Alzheimer’s disease (AD) and Parkinson’s disease (PD), and discussed therapeutic prospects and future study goals. Understanding these processes will promote the investigation of T-cell immunity in brain disorders and provide new intervention strategies for the treatment of brain injury and neurodegeneration.

## 1 Introduction

The incidence of brain injury and neurodegenerative diseases, which lead to the loss of specific neurons and dysfunction of neuronal networks resulting in impaired cognitive function, behavior, and motor functions, has increased worldwide in recent years (Carroll, 2019; Wareham et al., 2022). For many years, researchers have mainly focused on neurons to elucidate the mechanism of neuronal death, and it has only a few years since researchers have taken an interest in the roles of peripheral immune cells in brain injury. Upon brain injury, inflammation-mediated blood-brain barrier (BBB) damage leads to the recruitment of peripheral immune cells into the brain, which may directly interact with resident brain cells or indirectly release immune mediators to modulate the immune niche and ultimately affect the outcome of brain injury (Iadecola et al., 2020; Salvador and Kipnis, 2022). Similar to brain injury, immune cell-mediated inflammation is considered a hallmark of neurodegeneration. The neuroinflammatory responses mediated by innate and adaptive immunity indeed contribute to the progression of neurodegenerative diseases (NDDs) and regulate neuronal death (Ribeiro, 2023; Wilson et al., 2023). Thus, we summarized the role of CD8^+^ T cells in both brain injury and neurodegeneration to reveal the similarities and differences underlying the regulation of neuronal death and function which will provide insights into new ways to treat neurological diseases.

At present, it is well established that T cells are involved in brain homeostasis as well as neurological diseases (Ellwardt et al., 2016; Evans et al., 2019). The roles of CD4^+^ T cells in brain injury and neurodegenerative diseases have been summarized and discussed in many reviews (Iadecola and Anrather, 2011; Berriat et al., 2023). CD8^+^ T cells are a subpopulation of T lymphocytes that can differentiate into cytotoxic effector T cells when exposed to antigens. In addition to secreting tumor necrosis factor alpha (TNFα) and interferon (IFN)-γ to exert immune regulatory functions, CD8^+^ T cells can also directly release granzymes and perforins and upregulate the expression of FASL to trigger target cell death (Kaech and Cui, 2012; Reina-Campos et al., 2021). Considering their critical role and translational application in tumor therapies (Jiang et al., 2019; St Paul and Ohashi, 2020), understanding their functional properties and molecular mechanisms of the CD8^+^ T-cell populations in brain injury and neurodegeneration may facilitate the design of therapies to alleviate diseases.

In this review, we described the properties of CD8^+^ T-cell differentiation and function, summarized the roles of CD8^+^ T cells in brain injury, including ischemic stroke and traumatic brain injury (TBI), and neurodegenerative diseases, including Alzheimer’s disease (AD) and Parkinson’s disease (PD), and discussed future study goals.

## 2 Differentiation and function of CD8^+^ T cells

CD8^+^ T cells are generated in the thymus and act as important components of adaptive immunity, which play important roles in intracellular pathogen clearance and cancer (Kumar et al., 2018; Tabilas et al., 2023). Naïve CD8^+^ T lymphocytes can be activated by recognizing peptides presented by major histocompatibility complex (MHC) class I molecules. Under the coordinated activation of signals mediated by antigens, costimulatory molecules, and various cytokines, naïve CD8^+^ T cells undergo massive expansion and differentiation into various kinds of effector and memory subpopulations, which help to fight against pathogens and exert long-term protection (Mittrücker et al., 2014; Sun et al., 2023).

When CD8^+^ T cells encounter antigens in an acute inflammatory context, such as bacterial or viral infection, they differentiate into cytolytic effector T cells, also known as CD8^+^ cytotoxic T lymphocytes (CTLs). CD8^+^ CTLs can directly secrete granzymes and perforins and enhance the expression of Fas ligand (FASL) to trigger target cell death (Golstein and Griffiths, 2018). In addition, effector CD8^+^ T cells also secrete tumor necrosis factor α (TNFα) and interferon (IFN)-γ to exert immune functions (Kaech and Wherry, 2007). In tumor immunity, some CD8^+^ T cells exhibit an exhausted state and become dysfunctional, during which CD8^+^ T cells upregulate the expression of many inhibitory receptors, such as CTLA4, PD1, TIM3, and LAG3, and lose the ability to produce effector cytokines or cytotoxic molecules (Speiser et al., 2016; Philip and Schietinger, 2022). After pathogen or antigen clearance, most effector T cells die by apoptosis (Badovinac et al., 2004).

After exerting effector function, a small number of antigen-experienced CD8^+^ T cells survive and remain as memory CD8^+^ T cells, which can be rapidly reactivated and regain effector functions when re-exposed to antigens (Kaech and Cui, 2012). Currently, heterogeneous populations of CD8^+^ memory T-cell types have been defined according to their expression of surface markers or functional properties, including central memory (T_*CM*_) and effector memory CD8^+^ T cells (T_*EM*_), and tissue-resident memory (T_*RM*_) cells (Jameson and Masopust, 2009; Gerlach et al., 2010). CD8^+^ memory T cell types are identified mainly based on the expression of CD62L and CCR7; T_*CM*_ cells are mainly CD62L*^hi^*CCR7*^hi^* and T_*EM*_ cells exhibit CD62L*^low^*CCR7*^low^* phenotype. Unlike T_*CM*_ and T_*EM*_ cells, which continuously circulate in the peripheral blood (PB), T_*RM*_ cells mainly reside in the brain and mucosal tissues and have a characteristic of CD103*^hi^*CD69*^hi^* CD27*^low^* phenotype (Kaech and Wherry, 2007; Sheridan and Lefrançois, 2011).

The differentiation and function of CD8^+^ T cell subsets are orchestrated by transcription factors, epigenetic regulators, and metabolic programs at different tissues upon immune challenge (Kaech and Cui, 2012; Cao et al., 2023). After stimulation, the maintenance of memory CD8^+^ T cell relies on cytokines including IL-7 and IL-15, which contribute to cell survival and self-renewal of memory CD8^+^ T cell populations (Surh and Sprent, 2008). The roles of CD8^+^ T cells in tumor immunity and infectious diseases are well established (St Paul and Ohashi, 2020; Reina-Campos et al., 2021). Recently, more specific CD8^+^ T cell subpopulations in disease progression or tissue-specific regulation are under investigation with the development of single-cell RNA-sequencing (ScRNA-seq) technologies. Notably, the functions of CD8^+^ T cells in brain injury and neurodegeneration are largely unknown, which is the focus of this review.

## 3 CD8^+^ T cells in brain injury

Acute brain injuries such as ischemic stroke, hemorrhagic stroke and traumatic brain injury (TBI) and chronic autoimmune-induced brain injuries remain a major threat to human health (McKee and Daneshvar, 2015; Stampanoni Bassi et al., 2022). As many excellent reviews have summarized the well-established roles of CD8^+^ T cells in autoimmune-related multiple sclerosis (Sinha et al., 2015; Brummer et al., 2022), we only review the role of CD8^+^ T cells in acute brain injuries here. The common pathological aspect of these brain injuries is the occurrence of neuroinflammation (Jayaraj et al., 2019; Morganti-Kossmann et al., 2019). The released damage-associated molecular patterns (DAMPs) of dying cells trigger robust inflammatory responses within the brain that damage the BBB and lead to the infiltration of peripheral immune cells, such as neutrophils, monocytes/macrophages and T cells (Zhang Z. et al., 2022; Bersano et al., 2023). The role of CD8^+^ T cells in brain injury is being discovered.

### 3.1 Ischemic stroke

Ischemic stroke caused by intracranial vascular occlusion is a devastating brain injury with considerable mortality and morbidity worldwide. Intravenous alteplase and thrombectomy are not always effective owing to reperfusion-induced injury. Uncovering the underlying mechanism and alleviating brain damage remain the focus of research (Pan et al., 2007). Many reports have demonstrated the roles and mechanisms of different CD4^+^ T-cell subsets in both the acute injury phase and long-term functional recovery phase (Zhang Z. et al., 2022; Wang et al., 2023). Similar to CD4^+^ T cells, CD8^+^ T cells are also profoundly activated after ischemic stroke, and the accumulation of CD8^+^ T cells peaks approximately 3–4 days after stroke onset, with cell numbers decreasing overtime (Gelderblom et al., 2009), and of note many studies also showed its existence in the chronic phase (Xie et al., 2019; Ahnstedt et al., 2020). Therefore, CD8^+^ T cells may participate in different stages of ischemic stroke.

**FIGURE 1:**
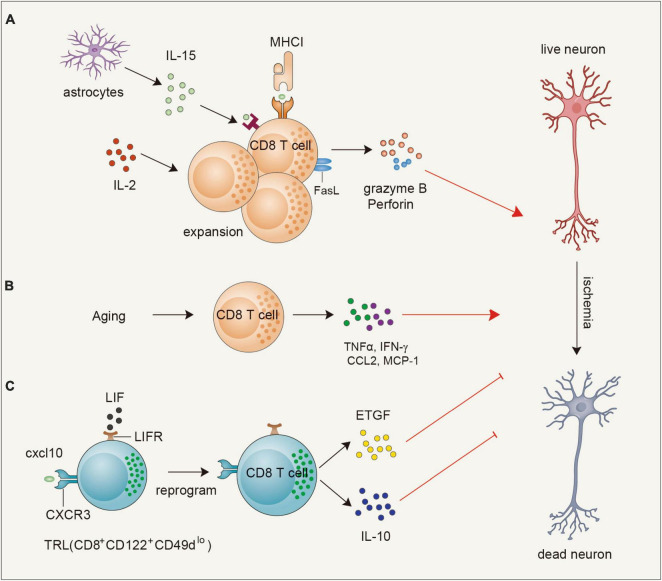
Functions and mechanisms of CD8^+^T cells in ischemic brain injury. **(A)** Astrocyte-derived IL-15 or endogenous IL-2 can promote the expansion and activation of CD8 + T cells to secrete granzyme B and perforin or upregulate FASL to exacerbate ischemic neuronal death. **(B)** During the aging process, CD8^+^ T cells are triggered to secrete TNFα, IFN-γ, CCL2, and MCP-1 to contribute to neuronal death. **(C)** A population of CD8^+^ T cells, namely T regulatory-like cells (TRL, CD8^+^CD122^+^ CD49d^lo^) infiltrate into the brain at early stages of ischemic stroke and are reprogrammed to upregulate leukemia inhibitory factor (LIF) receptor and induce epidermal growth factor-like transforming growth factor (ETGF), and interleukin 10 (IL-10) expression to exert neuroprotection through direct neuron protection or indirect anti-inflammatory effect. IL-2, interleukin 2; FASL, Fas ligand; TNFα, tumor necrosis factor alpha; IFN-γ, interferon-gamma; TRL, T regulatory-like cells; LIF, leukemia inhibitory factor; ETGF, epidermal growth factor-like transforming growth factor.

CD8^+^ T cells can both be detrimental and beneficial for acute ischemic brain injury (Figure 1). As cytotoxic lymphocytes, CD8^+^ T cells can exert a direct cytotoxic effect on neurons. One study showed that depleting CD8^+^ T cells by using a CD8α blocking antibody alleviates infarct volume and behavioral deficits in both transient middle cerebral artery occlusion (tMCAO) and permanent MCAO ischemia model mice. Correspondingly, adoptive transfer of CD8^+^ T cells into RAG1-knockout mice increases infarct size. In addition, the transfer of perforin-deficient CD8^+^ T cells reversed the detrimental effects, while IFNγ-knockout mice failed to alleviate the increased infarction, indicating perforin-mediated neurotoxicity of CD8^+^ T cells (Mracsko et al., 2014b). Strategies that increase CD8^+^ T-cell infiltration or cytotoxic function exacerbate ischemic brain injury (Li et al., 2017; Lee et al., 2018; Zhou et al., 2019; Fan et al., 2020). FASL enhances the cytotoxicity of CD8^+^ T cells to neurons after ischemic stroke. Inactivation of FASL on CD8^+^ T cells protects mice against neuronal death, which is mediated by compromised the expression of 3-phosphoinositide-dependent protein kinase-1 (PDPK1), a kinase responsible for the cytolytic effect of CD8^+^ T cells by regulating the phosphorylation of mTOR (Finlay et al., 2012; Fan et al., 2020). IL-15 potently induces the proliferation of memory CD8^+^ T cells in an antigen-independent manner and augments their effective function (Kim et al., 2008). Lee et al. (2018) showed that IL-15 blockade by using IL-15 knockout mice and an IL-15 blocking antibody reduces brain infarction. This neuroprotective effect is achieved by reducing the activation of CD8^+^ T cells and NK cells (Lee et al., 2018). Consistently, GFAP-driven IL-15 transgenic mice exhibit increased brain infarction compared with non-transgenic mice owing to the augmented accumulation of CD8^+^ T cells and NK cells (Li et al., 2017). Moreover, administration of an IL-2 neutralizing antibody alleviates brain infarction and promotes remyelination by limiting CD8^+^ T-cell infiltration and activation (Zhou et al., 2019). Therefore, blocking the function of cytokines or molecules that are required for the maintenance and the cytotoxicity of CD8^+^ T cells may be beneficial for the ischemic brain.

Risk factors for ischemic stroke may aggravate brain injury by regulating CD8^+^ T-cell functions. Perioperative ischemic stroke is one of the most severe complications of surgery and has severe public health implications (Vlisides and Mashour, 2016). Compared to sham mice, mice subjected to ileocecal resection showed an obvious increase in the number of CD44*^hi^*CD62L*^lo^*CD8^+^ T effector lymphocytes in peripheral and ischemic brain tissues at Day 7 after ischemic stroke, whereas the number of brain-infiltrating CD4^+^ T lymphocytes and neutrophils was not significantly different. Further mechanistic study demonstrated that immunometabolite S-2HG accumulates in CD8^+^ T cells in perioperative stroke mice, promotes proliferation and activation of CD8^+^ T lymphocytes, and exerts direct neurotoxicity (Zhang F. et al., 2022). Aging is another important risk factor contributing to ischemic stroke (Chen et al., 2010). In the aging brain, a population of CD8^+^ T cells are present in perivascular and parenchymal regions and exhibit a memory/effector phenotype with high T cell receptor (TCR) expression and show a positive correlation with anti-inflammatory phenotype of microglia which facilitates immune surveillance. However, when the aging mice are subjected to MCAO, these CD8^+^ T cells contribute to age-related exacerbation of acute ischemic brain injury instead of alleviating it by promoting the production of proinflammatory cytokines as well as recruitment of peripheral leukocytes (Ritzel et al., 2016). Therefore, CD8^+^ T cells may have opposing roles in homeostasis and ischemic stroke progression of the aging brain.

In addition to their detrimental effect, recent studies also discovered a subset of regulatory CD8^+^ T cells that can exert an early protective effect. Bodhankar et al. (2015) discovered that transfer of IL-10-positive B cells into MCAO mice at an early time point leads to the generation of a dominant regulatory Treg population (IL-10^+^ CD8^+^ CD122^+^) both in the ischemic brain and spleen. Coincidentally, Cai et al. (2022) showed that during the early stage of brain ischemia, the upregulated expression of CXCL10 interacts with CXCR3 on CD8^+^CD122^+^CD49d*^lo^* T regulatory-like cells (CD8^+^ TRLs) to increase their infiltration in the brain. Interestingly, these recruited CD8^+^ TRLs are reprogrammed to upregulate leukemia inhibitory factor (LIF) receptor and exert neuroprotection through direct neuronal protection via promoting the expression of epidermal growth factor-like transforming growth factor (ETGF), and indirect anti-inflammatory effect through increasing the production of interleukin 10 (IL-10) (Cai et al., 2022). Thus, transfer of this regulatory CD8 T-cell subset may offer new perspectives to protect the brain from acute injuries. In addition, with the rapid development of Sc-RNA-seq and cytometry by time-of-flight (CytOF) technologies (Zhang et al., 2020; Jovic et al., 2022), the characteristics and functions of various CD8^+^ T-cell subsets in the acute phase and chronic phase will be clarified in the future.

In addition to the important function of CD8^+^ T cells in the acute phase of ischemic stroke, the role of CD8^+^ T cells in the chronic phase is also beginning to be exposed. One study found that CD8^+^ T cells remain at a higher number in the chronic phase and worsen functional recovery by increasing the infiltration of other immune cells, such as B cells, neutrophils and monocytes, to promote neuroinflammation (Selvaraj et al., 2021). For many decades, multiple studies have shed light on the molecular mechanisms that regulate neurological recovery during the weeks after ischemic stroke (Carmichael, 2006; Cramer and Riley, 2008; Zhang Z. et al., 2022). Considering the existence of CD8^+^ T cells during the chronic recovery phase of ischemic stroke, it will be interesting to reveal how these sustained CD8^+^ T cells communicate with brain-resident cells to regulate functional aspects of recovery such as neurogenesis, oligodendrogenesis and neuronal regeneration in the long term. In addition, what signal determines whether CD8^+^ T cells exert a protective effect or detrimental effect in homeostasis or during disease progression remains unknown.

### 3.2 Hemorrhagic stroke

Hemorrhagic stroke which includes the subtypes of intracerebral hemorrhage (ICH) and subarachnoid hemorrhage (SAH) has considerable morbidity and mortality rates. Similar to ischemic stroke, inflammation and the host immune response contribute to the pathophysiology of hemorrhagic stroke (Montaño et al., 2021; Nasa, 2022; Ohashi et al., 2023). After hemorrhagic stroke, the BBB is damaged and peripheral immune cells infiltrate into the brain. The function of peripheral infiltrated immune cells in hemorrhagic stroke has been studied, although less than ischemic stroke (Mracsko et al., 2014a; Li and Chen, 2023). Previous studies have observed the increase of CD8^+^ T lymphocytes in the early phase of ischemic stroke and last for 21 or 28 days (Xue and Del Bigio, 2003; Mracsko et al., 2014a). Considering the regulatory role of CD8^+^ T cells in ischemic stroke, the function of CD8^+^ T cells in hemorrhagic stroke needs investigation in the future.

### 3.3 Traumatic brain injury

Traumatic brain injury (TBI) remains a substantial cause of morbidity and mortality in both adults and children. TBI involves complex neurological processes, including acute molecular changes and long-term neurocognitive sequelae (Bramlett and Dietrich, 2015; Taylor et al., 2017). Several studies have revealed the involvement of T cells in both the acute phase and chronic phase of TBI (Bao et al., 2021; Xu et al., 2021). Compared with the study of CD4^+^ T cells in ischemic stroke, the study of CD8^+^ T cells in TBI is still limited.

**FIGURE 2:**
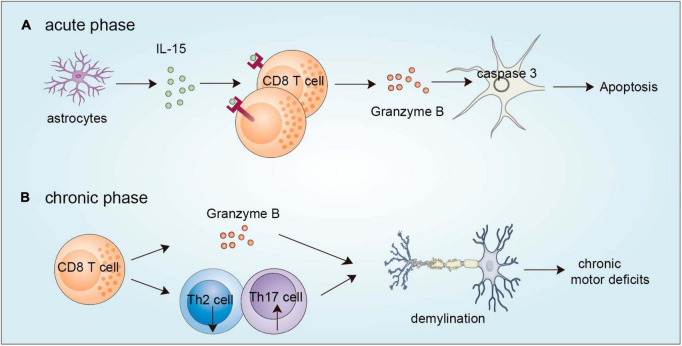
Functions of CD8^+^ T cells in TBI. **(A)** During the acute phase, astrocytes are activated to produce IL-15, which evokes CD8^+^ T-cell activation to release granzyme B, which in turn acts on neurons and leads to caspase 3-induced apoptosis. **(B)** During the chronic phase, brain-infiltrating CD8^+^ T cells can secrete granzyme B and regulate Th2/Th17 shift to accelerate demyelination. TBI, traumatic brain injury.

CD8^+^ T cells can infiltrate the brain and accumulate in the TBI site (Ling et al., 2006). CD8^+^ T cells exert a detrimental effect via their cytotoxic effect in the acute damage stage. By using a model of TBI, Ling et al. (2006) observed that astrocytes are activated and produce IL-15, which triggers CD8^+^ T-cell activation and the release of granzyme B. The released granzyme B can in turn act on neurons and induce neuronal apoptosis by caspase-3-induced PARP cleavage (Wu et al., 2021). Pituitary adenylate cyclase activating polypeptide can protect mice from TBI-induced injury by balancing CD4^+^ and CD8^+^ T-cell ratios and functions (Hua et al., 2012). In addition to aggravating acute brain injury, traumatic brain injury (TBI) also results in myelin-related pathology and long-term disabilities in many survivors. At 8 weeks after TBI, the number of effector/memory CD8^+^ T cells is increased in the injured brain and these CD8^+^ T cells release granzyme B, which precedes Th17 cell infiltration, and is associated with an elevated autoantibody response and progressive impairment of neurological functions. Genetic deletion of CD8^+^ T cells by using β2-microglobulin-deficient mice or pharmacological depletion of CD8^+^ T cells by using a CD8^+^ blocking antibody improves neurological outcomes and produces a neuroprotective Th2/Th17 immunological shift. However, the deletion of CD4^+^ T cells does not have this effect, and the depletion of B cells results in even more severe neurological dysfunction, demonstrating the specific effects of CD8^+^ T cells in regulating long-term functional impairment after TBI (Daglas et al., 2019). Despite the above reports of CD8^+^ T cells in the acute injury phase and chronic recovery phase of TBI (Figure 2), more questions still exist. For example, do CD8^+^ T cell populations function differently during TBI progression? Can CD8^+^ T cells directly interact with brain-resident cells to affect TBI progression? What is the difference in the cytokine profile of CD8^+^ T cells during different stages of TBI? Which factors recruit CD8^+^ T cells into the brain after TBI? Resolving these questions may provide important insights to increase the efficiency of TBI treatment using immunotherapy.

Like ischemic stroke, TBI also induces peripheral immunosuppression which is detrimental, as it increases the incidence of hospital-acquired infections (HAIs) (Ritzel et al., 2018; Sribnick et al., 2022). Mechanistically, this peripheral immunosuppression has been attributed to a disturbance in the well-balanced bidirectional communication between the brain and the immune system owing to injury (Hazeldine et al., 2015; Sribnick et al., 2022). One study showed that TBI induces the activation of the sympathetic nervous system, which triggers the expression of PD-1, a marker of T-cell exhaustion, on CD4^+^ and CD8^+^ T cells leading to immunosuppression. Inhibition of sympathetic nervous system by propranolol reverses this effect *in vivo* (Yang et al., 2019). More studies are needed to gain a better understanding of the mechanisms underlying TBI-induced changes in the immune system and how to manipulate these changes to treat hospital-acquired infections after TBI.

Notably, it is important to be aware of the specific staining of CD8^+^ T cells when investigating CD8^+^ T cells as one study found that a population of macrophage/microglia also expresses CD8 after TBI and localizes in the border of the pannecrosis, which may have a role in lesion development after TBI (Zhang et al., 2006). How to distinguish these cells to avoid false conclusions should be given immediate attention although why other immune cells also express CD8 remains unknown.

### 3.4 Other kinds of brain injuries

In addition to acute stroke-induced brain injury and TBI, other kinds of less frequent brain injuries also exist. Radiation-induced brain injury (RIBI) remains one of the most common medical complications of brain radiation therapy. In addition to the acute adverse effects of radiation exposure, cranial radiotherapy also results in severe and delayed onset of brain injury as well as cognitive impairment. Uncontrolled progressive development of brain lesions can eventually result in cerebral herniation and even death (Ali et al., 2019). However, the underlying mechanism of delayed RIBI is poorly understood. Notably, peripheral lymphocytes infiltration has been reported as a common feature in the necrotic brain area of patients with RIBI (Kureshi et al., 1994; Yoritsune et al., 2014), suggesting the involvement of adaptive immunity in the development of RIBI. Recently, one study found that CD8^+^ T cells infiltrate and expand in the lesioned brain tissue of RIBI patients by using Sc-RNA-seq and T-cell receptor sequencing techniques. In addition, these infiltrated T cells are in an activation state as granzyme B and perforin are secreted from cytotoxic CD8^+^ T cells in the CSF of RIBI patients. Further mechanistic studies showed that microglia-derived chemokines CCL2/CCL8 mediate the infiltration of CCR2^+^/CCR5^+^ CD8^+^ T cells in the brain parenchyma. Inhibition of CCL2 and CCL8 function by conditional knockout mice and neutralization antibodies alleviate RIBI as well as ischemic brain injury (Kumari and Gensel, 2023; Shi et al., 2023). This study reveals the participation of microglial-mediated activation of CD8^+^ T cells in brain damage and provides direct evidence of brain-immune communications in the regulation of RIBI.

Perinatal brain injury influences infants born at gestational ages and may lead to substantial long-term neurodevelopmental impairment, including cognitive, neurological, sensory and motor disabilities (Novak et al., 2018; Reiss et al., 2022). Perinatal brain injury can be triggered by hypoxia-induced ischemia, maternal infection or inflammation that elicits a cascade of events potentiating perinatal brain injury (Novak et al., 2018; Leavy and Jimenez Mateos, 2020). Several studies found an increase in CD8^+^ T cell numbers in the placenta following maternal intrauterine inflammation (IUI). Maternal CD8 T-cell-depletion or maternal administration of bone marrow-derived mesenchymal stem cells (BMMSCs) results in a decreased number of placental CD8^+^ T cells which reduces perinatal brain injury and improves neurological outcomes in the offspring (Lei et al., 2018; Zhao et al., 2020). Future studies are needed to reveal neuroinflammatory processes and design therapeutic strategies to alleviate perinatal brain injury.

## 4 CD8^+^ T cells in neurodegeneration

Neurodegenerative diseases (NDDs), such as Alzheimer’s disease (AD), Parkinson’s disease (PD), and amyotrophic lateral sclerosis (ALS) are characterized by the processive accumulation of protein aggregates and the loss of neurons in specific brain regions. For example, in AD, the aggregates are intracellular Tau and extracellular Aβ and hippocampal as well as cortical neurons are impacted. In PD, the alpha-synuclein and TDP-43 are intracellular aggregates and the neurons in the midbrain substantia nigra are impacted (Dugger and Dickson, 2017; Berriat et al., 2023). For many years, researchers have focused on neurons to reveal the underlying mechanism of neuronal loss. Only in the past few years, has multiple pieces of evidence revealed the critical role of peripheral immune cells in the regulation of the pathogenesis of NDDs. Immune cell-mediated inflammation is now considered a hallmark of NDDs (Wilson et al., 2023). However, whether immune cell-mediated inflammation is a consequence or cause of PD remains unknown (Appel, 2012). It is widely accepted that the neuroinflammatory responses mediated by innate and adaptive immunity indeed contribute to the progression of NDDs and mediate neuronal death. As an important subpopulation of T cells, the role of CD8^+^ T cells in NDDs is being recognized.

### 4.1 Alzheimer’s disease (AD)

In patients with AD, the immune cell diversity and activation states are altered, indicating their involvement in the progression of AD. Early studies suggested a decreased proportion of CD8^+^ T cells in AD patients (Pirttilä et al., 1992; Shalit et al., 1995), and some observed low expression of the costimulatory molecules CD28 (Speciale et al., 2007). However, other studies have found no significant changes in CD8^+^ T-cell frequency in the peripheral blood between mild AD patients and healthy controls (Larbi et al., 2009). These contrasting results may result from samples collected from patients in different stages of AD and testing methods used, as some macrophages and NK cells are also observed to express CD8 in some conditions (Popovich et al., 2003; McKinney et al., 2021). Thus, researchers should be cautious when testing the existence of CD8^+^ T cells, and add other co-expression markers of T cells to draw a solid conclusion. With the development of mass cytometry and ScRNA-seq technologies, some studies have observed an increase in CD8^+^ T cells numbers in the peripheral blood (PB) and CSF of AD patients and characterized the phenotype of CD8^+^ T cells (Schindowski et al., 2007; Lueg et al., 2015; Gate et al., 2020). Gate et al. (2020) found an increased number of CD3^+^CD8^+^CD27^–^ T effector memory CD45RA^+^ (TEMRA) cells in the mononuclear cells of peripheral blood by using mass cytometry. These cells are negatively associated with cognitive function. In addition, the CD8^+^ T cells in the cerebrospinal fluid of AD patients are clonally expanded and antigen-specific cells (Gate et al., 2020; Heneka, 2020; McManus and Heneka, 2020). This study raises the question of what antigen brain-infiltrating CD8^+^ T cells recognize and mediate its clonal expansion. Interestingly, Gate et al. (2020) found some Epstein-Barr virus (EBV)-specific clones of CD8^+^ T cells, although they are not highly expanded clones. EBV DNA is present in approximately six percent of AD brains (Carbone et al., 2014). In addition to EBV, cytomegalovirus (CMV) is a member of the herpesvirus family that is prevalent throughout the world. Approximately seventeen percent of patients with AD are positive for human herpes virus (Carbone et al., 2014). A reduced frequency of CMV-specific CD8^+^ T cells is present in AD patients compared with other CMV-positive patients (Westman et al., 2013). Based on this evidence, we cannot define the direct relationship between viral infections and the progression of AD, as virus-induced immune response is commonly present in elderly people. Future studies are needed to investigate whether other antigen-specific T cell clones exist and participate in the pathogenesis of AD. Interestingly, in PD patients, peptides derived from α-synuclein can be presented to CD8^+^ T cells and leads to its activation (Sulzer et al., 2017; Hobson and Sulzer, 2022). Whether Aβ can generate peptides to specifically activate CD8^+^ T cells needs further investigation. In addition, if antigen specific CD8^+^ T cells are identified, it will be interesting to further test whether they are derived in the brain or in the peripheral immune system. Comparing the antigen specificity of T cells may help identify some T cell specific subpopulations which may benefit both diagnosis and treatment.

**FIGURE 3:**
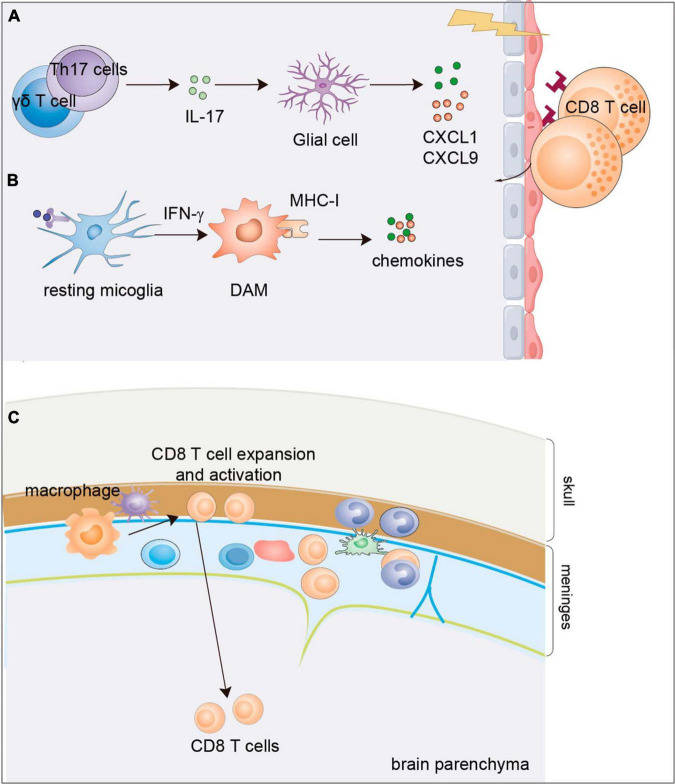
Mechanisms of CD8^+^ T-cell infiltration into the brain parenchyma of AD. **(A)** IL-17 cytokine possibly produced by Th17 cells or γδ T cells was observed to express in the cortex and hippocampus of APP/PS1 mice which enhances the production of chemokines CXCL1 and CXCL9/10 from the glial cells to trigger the infiltration of CD8^+^ T lymphocyte. **(B)** Disease associated microglia can be induced by cytokines such as IFN-γ to release chemokines and cytokines to recruit CD8^+^ T cells. **(C)** Activation of meningeal CD8^+^ T cells by meningeal macrophage facilitates their infiltration into the parenchyma. DAM, disease associated macroglia; AD, Alzheimer’s disease.

In addition to the alteration of CD8^+^ T cells in the PB and CSF, the presence of CD8^+^ T cells is also observed in the brains of both AD patients (Itagaki et al., 1988; Merlini et al., 2018; Unger et al., 2020) and AD model mice (Laurent et al., 2017; Unger et al., 2020; Michael et al., 2021; Fernando et al., 2023). The substantial infiltration of CD8^+^ T cells in the brain raises a question of what signal mediates CD8^+^ T cell infiltration into the brain. One study indicated that pro-inflammatory cytokine IL-17 may mediate the enrichment of CD8^+^ T cells. IL-17 was expressed in the cortex and hippocampus of APP/PS1 model mice. IL-17 enhances the production of chemokines CXCL1 and CXCL9/10 from glial cells to trigger the infiltration of myeloid cells and CD8^+^ T lymphocytes (Ye et al., 2023). Then, comes another question. Which cells release IL-17? As Aβ immunization promotes IL-17 production by T helper 17 (Th17) cells (McQuillan et al., 2010; Zhang et al., 2013), one possibility is that the upregulated expression of IL-17 may result from elevated Th17 cell numbers. In addition, γδ T cells are regarded as one of the bridges connecting innate and adaptive immunity, and they can quickly secrete cytokines, especially IL-17 and IFNγ, after activation (Bonneville et al., 2010). More importantly, a subset of IL-17-producing γδ T cells populates the meninges in normal brains (Ribeiro et al., 2019). These evidence indicate that γδ T cells may also contribute to IL-17 production in the brain to incite neurodegeneration as an early response. In addition, brain-resident microglia also release chemokines and cytokines to recruit CD8^+^ T cells. In human and mouse brains with tauopathy, parenchymal microglia exhibit a disease-associated phenotype with increased expression levels of MHC-I and inflammatory chemokines and cytokines, which promote the recruitment of peripheral T cells into the parenchyma and drive neurodegeneration (Chen et al., 2023). The important role of meningeal immunity is now being recognized. One study provides evidence that the activation of meningeal CD8^+^ T cells facilitates their infiltration into the parenchyma. CD8^+^ T cells are highly activated in the meninges and brains of patients with NDDs, and undergo clonal expansion which may further potentiate the infiltration of CD8^+^ T cells into the parenchyma and exacerbate brain degeneration (Hobson et al., 2023; Figure 3).

CD8^+^ T cells regulate the progression of AD (Table 1). One study used a CD8 blocking antibody to deplete CD8^+^ T cells in APP-PS1 mouse models, and neither cognitive outcome nor plaque pathology was altered after depletion of CD8^+^ T cells. However, the expression of several neuronal and synapse-related genes, including activity-regulated cytoskeleton-associated protein (Arc) and neuronal PAS domain protein 4 (Npas4) was altered in the hippocampus in the presence of a CD8 blocking antibody (Unger et al., 2020). In addition, CD8^+^ T cells are present in the hippocampus of tau transgenic mice and the cortex of patient brains with P301L tau mutation. CD3 blocking antibody inhibits T-cell infiltration in the tau transgenic mice and improves spatial memory impairment (Laurent et al., 2017). Administration of the leukotriene receptor antagonist montelukast (MTK) in 5XFAD mice improves cognitive function. Mechanistically, MTK treatment increases the number of Tmem119^+^ microglia and decreases the expression level of AD-associated and lipid droplet accumulation-related genes, which reduces infiltration of CD8^+^ T cells into the brain parenchyma (Michael et al., 2021). Therefore, factors or molecules that can specifically modulate CD8^+^ T-cell expansion and function can be evaluated in AD models. Interestingly, a disease-associated CD8^+^ T-cell population is present in the aged AD mouse brains on a 5XFAD background. These cells express high levels of immune-responsive genes (ISGs) at the transcriptional and epigenetic levels, which are normally induced by interferons (IFNs) (Fu et al., 2023). Depletion of CD8^+^ T cells by using a β2m-deficient mouse leads to a reduction in amyloid-β plaque burden and improves spatial memory in AD model mice (Fernando et al., 2023).

**TABLE 1:** Evidence of CD8^+^T cells in neurodegeneration.

Neurodegenerative diseases		Main evidence of CD8^+^ T cell infiltration and effects on neurodegeneration
AD	Infiltration	PB or CSF of AD patients: decreased proportion (Shalit et al., 1995; Speciale et al., 2007; Larbi et al., 2009); no difference (McKinney et al., 2021); increased number (Schindowski et al., 2007; Gate et al., 2020; Heneka, 2020); increased number of effector/memory CD8^+^ T cells (Heneka, 2020). Brain tissue of AD patients: present in the brain (Itagaki et al., 1988; Merlini et al., 2018; Hobson and Sulzer, 2022). Mice model: accumulated in the mouse brain (Laurent et al., 2017; Merlini et al., 2018; Unger et al., 2020; Michael et al., 2021)
AD	Effects	Blocking CD8 no alternation of cognitive outcome or plaque pathology (Merlini et al., 2018); Blocking CD3 improves spatial memory (Unger et al., 2020); β2m-deficiency alleviates AD (Fernando et al., 2023); β2m-deficiency exacerbates AD (Su et al., 2023).
PD	Infiltration	PB or CSF of PD patients: CD4 to CD8 ratio changes (He et al., 2022; Fu et al., 2023); decreased number (Baek et al., 2016); CD8^+^ T cell terminal effector phenotype (Marsh et al., 2016), with high expression of TNFα (Wang P. et al., 2021; Heming et al., 2022) and show immunosenescence (Hisanaga et al., 2001; Chen et al., 2021). Brain tissue of PD patients: significant infiltration in the SN (Williams-Gray et al., 2018; Kouli et al., 2021; Jiang et al., 2023). Mice model of PD: present in the PD brains (Brochard et al., 2009; Galiano-Landeira et al., 2020).
PD	Effects	MHC-I knockdown suppresses dopaminergic neuronal loss (Thakur et al., 2017); RAG-KO alleviates 6-OHDA-induced PD (Williams et al., 2023); CD8^+^ T cells depletion prevents colitis-induced suppression of dopaminergic markers (Ip et al., 2015).
ALS	Infiltration	PB or CSF of ALS patients: show a clonally expanded effector memory CD8^+^ T cells (Shi et al., 2007; Fiala et al., 2010; Houser et al., 2021; Campisi et al., 2022; Yazdani et al., 2022), secrete increased granzyme B, IL-17, and IL-13 (Shi et al., 2007; Fiala et al., 2010; Yazdani et al., 2022). Mice model of ALS: present in the ALS brains (Campisi et al., 2022).
ALS	Effects	Depletion of CD8 T cell promotes the survival of motor neurons (Nardo et al., 2018; Coque et al., 2019).

Previous studies on the effects of T cells suggest that T cells may also have beneficial effects on the clearance of Aβ aggregates and improve cognitive behavior. Recently, one study showed that depletion of CD8^+^ T cells using a β2m-deficient mouse or impairing the accumulation or clonal expansion of CD8^+^ T cells by CCR6-deficiency increased Aβ deposition and cognitive impairment in 5XFAD mice by restraining the proinflammatory response of microglia (Su et al., 2023), which is in contrast with Fernando et al. (2023) study that also used β2m-deficient 5XFAD mice. In addition, the adoptive transfer of regulatory T cells into 3xTg transgenic AD mice reduces Aβ aggregate deposition and improves cognitive deficits by decreasing inflammatory cytokine production and microglial activation (Baek et al., 2016). Mice on a 5XFAD background with lymphocytes deficiency (RAG2-KO) exhibited greater Aβ deposition than that in control mice as a result of a reduction in microglia-mediated phagocytosis (Marsh et al., 2016). The studies indicate the complex and context-dependent roles of CD8^+^ T cells in the pathogenesis of AD. Strategies aimed at modulating CD8^+^ T-cell function to treat AD should distinguish the beneficial and detrimental roles of different CD8^+^ T cell subpopulations.

### 4.2 Parkinson’s disease (PD)

Similar to AD, many studies have reported an alteration in T lymphocytes in the peripheral blood (PBs) and CSF of PD patients (Baba et al., 2005; Wang P. et al., 2021; He et al., 2022; Heming et al., 2022). Early studies showed an increased or decreased ratio of CD4 to CD8^+^ T cells in the PBs and CSF of PD patients (Hisanaga et al., 2001; Baba et al., 2005). Further mapping indicates that the number of naïve CD8^+^ T cells is reduced (He et al., 2022) and that a large population of CD8^+^ T cells continuously progress from central memory to terminal effector T cells in the blood and CFS of PD patients based on single-cell transcriptome expression combined with TCR-sequencing (Wang P. et al., 2021). Moreover, these CD8^+^ T cells are activated and show high expression of TNFα (Chen et al., 2021; Jiang et al., 2023). During the early stages of PD, the population of terminally differentiated effector CD8^+^ TEMRA T cells, the hallmarks of immunosenescence, in peripheral blood is reduced and PD patients exhibits downregulated expression of senescence-related markers such as p16^*INK*4*a*^ compared with age and sex-matched healthy controls (Williams-Gray et al., 2018; Kouli et al., 2021). Whether immunosenescence contributes to the development and progression of PD deserves further investigation.

Evidence of the presence of CD8^+^ T cells in the brain is also increasing. CD8^+^ T cells infiltrate the substantia nigra pars compacta (SNpc) in patients during the early stage of PD before α-synuclein aggregation, which leads to neuronal death and synucleinopathy (Brochard et al., 2009; Galiano-Landeira et al., 2020; Halliday, 2020). Moreover, CD4 and CD8^+^ T cells are also present in the brains of PD model mice (Thakur et al., 2017). In an age-dependent PD model mice with genetic A30P/A53T double-mutated α-synuclein, infiltrated CD8^+^ and CD4^+^ T cells are correlated with the loss of dopaminergic neurons in the late stage of PD progression. A high proportion of infiltrated CD8^+^ T cells are tissue-resident memory T cells that express cytolytic granzymes and proinflammatory cytokines (Rauschenberger et al., 2022). This poses the question of how CD8^+^ T cells are activated in the brain, and what antigens they recognize.

Increasing evidence indicates that neurons can express MHC-I to promote antigen presentation to CD8^+^ T lymphocytes and leads to its activation. The neurotoxin MPTP induces oxidative stress in dopaminergic neurons which further upregulates the expression of MHC-I. The presentation of MHC-I is positively correlated with the number of CD8^+^ T cells (Wang B. Y. et al., 2021). Another *in vitro* study showed that MHC-I molecules can be induced in neurons by IFNγ released from microglia, activated by neuromelanin or alpha-synuclein, which present antigens to CD8^+^ T cells and mediate neurotoxicity (Cebrián et al., 2014). The antigens recognized by CD8^+^ T cells have been extensively studied. Currently, it is accepted that peptides can be derived from α-synuclein in PD patients, which is then displayed by MHC-I and activates CD8 cytotoxic T cell responses (Sulzer et al., 2017; Hobson and Sulzer, 2022). In addition, one study found that the response of CD4^+^ T cells and CD8^+^ T cells in the PBMCs from PD patients or healthy controls to common antigens from human pathogens showed no obvious difference (Williams et al., 2023). Therefore, the altered T cell response observed in PD patients may be induced via the recognition of autoantigens like α-synuclein rather than common antigens from foreign pathogens.

Interfering with CD8^+^ T-cell function affects the progression of PD (Table 1). Depletion of T cells by using RAG-KO mice exerts a protective effect on the 6-hydroxydopamine (6- OHDA)-induced PD model mice (Ip et al., 2015). Moreover, knockdown of MHC-I in neurons of the substantia nigra reduces the infiltration of CD8^+^ T cells and suppresses the loss of dopaminergic neurons (Cebrián et al., 2014). With the understanding of gut-brain interactions in the pathogenesis of brain diseases, one study explored whether colitis promotes the neuropathology of PD. Colon biopsies from PD patients exhibit high expression of inflammatory indicators Cd8b and NF-κB p65 and low expression of regulator of G-protein signaling 10 (RGS10), an inhibitor of NF-κB. Moreover, an obvious reduction in tyrosine hydroxylase levels and increased CD8^+^ T-cell infiltration and activation were observed in mice with experimental colitis. Experimental colitis-induced suppression of dopaminergic markers is recovered by depletion of CD8^+^ T cells indicating the important regulation of CD8^+^ T cells in mediating colitis-induced neuropathology (Houser et al., 2021). Investigation of the role of the gut-brain axis in other NDDs may provide common strategies for the treatment of NDDs.

### 4.3 Amyotrophic lateral sclerosis (ALS)

Similar to AD and PD patients, researchers discovered a clonally expanded effector memory CD8^+^ T- cell population in the peripheral blood and CSF of ALS patients (Campisi et al., 2022; Yazdani et al., 2022) and ALS animal models with mutations in the senataxin gene (SETX) (Carroll, 2019; Campisi et al., 2022). In the peripheral blood of ALS patients, an increased number of CD8^+^ T cell effector/memory cells was observed and these CD8^+^ T cells secreted increased granzyme B, IL-17 or IL-13 (Shi et al., 2007; Fiala et al., 2010; Kaur et al., 2022). Moreover, depletion of CD8^+^ T cells in MHC-I-deficient mice promotes the survival of motor neurons (Nardo et al., 2018; Coque et al., 2019; Table 1). The role of CD8^+^ T cells in other neurodegenerative diseases needs further investigation.

## 5 Conclusion and perspectives

T lymphocytes contribute to the maintenance of brain homeostasis and are involved in neurological diseases. As a special population of cytotoxic T lymphocytes, the function of CD8^+^ T cells in neurological disorders is being revealed. In this review, we mainly summarize evidence of the functions and molecular mechanisms of CD8^+^ T cells in brain injury as well as neurodegenerative diseases which all involve neuroinflammation and neuronal death, and discuss their therapeutic potentials and future questions. It is well established that CD8^+^ T cells infiltrate into the brain parenchyma after brain injury and neurodegeneration by responding to chemokines released by glial cells. It is interesting that some studies showed the significant infiltration prior to protein aggregates formation which indicates the involvement of CD8^+^ T cells in early phase of NDDs. The role of CD8^+^ T cells in brain injury and neurodegeneration seems to be more detrimental than beneficial, although a subset of regulatory CD8^+^ T cells was found to be protective in ischemic stroke and several reports indicate the protective role of T cells in promoting the clearance of protein aggregates in NDDs. These studies indicate the complex and context-dependent roles of CD8^+^ T cells in brain injury and neurodegeneration. Therefore, to avoid side effects, researchers should identify distinct CD8^+^ T cell subsets in the brain during disease progression, elucidate their respective roles and then design specific therapeutic strategies. In addition, compared with neurons and glial cells, CD8^+^ T cells are easy to detect and manipulate and are relatively accessible. However, their cytotoxic functions-mediated by granzymes and perforins and regulatory immune functions-mediated by IFNγ are not always exert the same effects on brain injury or neurodegeneration. To promote the application of CD8^+^ T cells-mediated immunotherapy in brain injury and NDDs, more thorough investigations are needed.

Future outstanding questions:

(1) What type of CD8^+^ T-cell subset is present and what are its functions during different stages of brain injury?
(2) Is there any specific feature of CD8^+^ T-cell subset exist during brain injury and neurodegeneration?
(3) How do CD8^+^ T-cell number and function change during the different stages of NDDs and affect the outcome of the disease, especially before severe protein aggregation?
(4) What antigens trigger CD8^+^ T cell activation during brain injury and neurodegeneration?
(5) What is the impact of the alternations in peripheral CD8^+^ T cells that do not infiltrate the brain on the progression of brain injury and NDDs?
(6) How do meningeal CD8^+^ T cells contribute to brain injury and NDDs?

## Author contributions

ZZ: Conceptualization, Funding acquisition, Software, Writing – original draft. ZD: Software, Writing – original draft. YC: Conceptualization, Funding acquisition, Investigation, Supervision, Writing – review and editing.

## References

[B1] Ahnstedt H. Patrizz A. Chauhan A. Roy-O’Reilly M. Furr J. W. Spychala M. S. (2020). Sex differences in T cell immune responses, gut permeability and outcome after ischemic stroke in aged mice. *Brain Behav. Immun.* 87 556–567. 10.1016/j.bbi.2020.02.001 32058038 PMC7590503

[B2] Ali F. S. Arevalo O. Zorofchian S. Patrizz A. Riascos R. Tandon N. (2019). Cerebral radiation necrosis: Incidence, pathogenesis, diagnostic challenges, and future opportunities. *Curr. Oncol. Rep.* 21:66. 10.1007/s11912-019-0818-y 31218455

[B3] Appel S. H. (2012). Inflammation in Parkinson’s disease: Cause or consequence? *Mov. Disord.* 27 1075–1077. 10.1002/mds.25111 22806694

[B4] Baba Y. Kuroiwa A. Uitti R. J. Wszolek Z. K. Yamada T. (2005). Alterations of T-lymphocyte populations in Parkinson disease. *Parkinsonism Relat. Disord.* 11 493–498. 10.1016/j.parkreldis.2005.07.005 16154792

[B5] Badovinac V. P. Porter B. B. Harty J. T. (2004). CD8+ T cell contraction is controlled by early inflammation. *Nat. Immunol.* 5 809–817. 10.1038/ni1098 15247915

[B6] Baek H. Ye M. Kang G. H. Lee C. Lee G. Choi D. B. (2016). Neuroprotective effects of CD4+CD25+Foxp3+ regulatory T cells in a 3xTg-AD Alzheimer’s disease model. *Oncotarget* 7 69347–69357. 10.18632/oncotarget.12469 27713140 PMC5342482

[B7] Bao W. Lin Y. Chen Z. (2021). The peripheral Immune System and traumatic Brain Injury: Insight into the role of T-helper cells. *Int. J. Med. Sci.* 18 3644–3651. 10.7150/ijms.46834 34790036 PMC8579286

[B8] Berriat F. Lobsiger C. S. Boillée S. (2023). The contribution of the peripheral immune system to neurodegeneration. *Nat. Neurosci.* 26 942–954. 10.1038/s41593-023-01323-6 37231108

[B9] Bersano A. Engele J. Schäfer M. K. E. (2023). Neuroinflammation and brain disease. *BMC Neurol.* 23:227. 10.1186/s12883-023-03252-0 37308838 PMC10258999

[B10] Bodhankar S. Chen Y. Lapato A. Vandenbark A. A. Murphy S. J. Saugstad J. A. (2015). Regulatory CD8(+)CD122 (+) T-cells predominate in CNS after treatment of experimental stroke in male mice with IL-10-secreting B-cells. *Metab. Brain Dis.* 30 911–924. 10.1007/s11011-014-9639-8 25537181 PMC4481189

[B11] Bonneville M. O’Brien R. L. Born W. K. (2010). Gammadelta T cell effector functions: A blend of innate programming and acquired plasticity. *Nat. Revi. Immunol.* 10 467–478. 10.1038/nri2781 20539306

[B12] Bramlett H. M. Dietrich W. D. (2015). Long-term consequences of traumatic brain injury: Current status of potential mechanisms of injury and neurological outcomes. *J. Neurotrauma* 32 1834–1848. 10.1089/neu.2014.3352 25158206 PMC4677116

[B13] Brochard V. Combadière B. Prigent A. Laouar Y. Perrin A. Beray-Berthat V. (2009). Infiltration of CD4+ lymphocytes into the brain contributes to neurodegeneration in a mouse model of Parkinson disease. *J. Clin. Invest.* 119 182–192. 10.1172/JCI36470 19104149 PMC2613467

[B14] Brummer T. Zipp F. Bittner S. (2022). T cell-neuron interaction in inflammatory and progressive multiple sclerosis biology. *Curr. Opin. Neurobiol.* 75:102588. 10.1016/j.conb.2022.102588 35732103

[B15] Cai W. Shi L. Zhao J. Xu F. Dufort C. Ye Q. (2022). Neuroprotection against ischemic stroke requires a specific class of early responder T cells in mice. *J. Clin. Invest.* 132:e157678. 10.1172/JCI157678 35912857 PMC9337834

[B16] Campisi L. Chizari S. Ho J. S. Y. Gromova A. Arnold F. J. Mosca L. (2022). Clonally expanded CD8 T cells characterize amyotrophic lateral sclerosis-4. *Nature* 606 945–952. 10.1038/s41586-022-04844-5 35732742 PMC10089623

[B17] Cao J. Liao S. Zeng F. Liao Q. Luo G. Zhou Y. (2023). Effects of altered glycolysis levels on CD8(+) T cell activation and function. *Cell Death Dis.* 14:407. 10.1038/s41419-023-05937-3 37422501 PMC10329707

[B18] Carbone I. Lazzarotto T. Ianni M. Porcellini E. Forti P. Masliah E. (2014). Herpes virus in Alzheimer’s disease: Relation to progression of the disease. *Neurobiol. Aging* 35 122–129. 10.1016/j.neurobiolaging.2013.06.024 23916950

[B19] Carmichael S. T. (2006). Cellular and molecular mechanisms of neural repair after stroke: Making waves. *Ann. Neurol.* 59 735–742. 10.1002/ana.20845 16634041

[B20] Carroll W. M. (2019). The global burden of neurological disorders. *Lancet Neurol.* 18 418–419. 10.1016/S1474-4422(19)30029-8 30879892

[B21] Cebrián C. Zucca F. A. Mauri P. Steinbeck J. A. Studer L. Scherzer C. R. (2014). MHC-I expression renders catecholaminergic neurons susceptible to T-cell-mediated degeneration. *Nat. Commun.* 5:3633. 10.1038/ncomms4633 24736453 PMC4024461

[B22] Chen R. L. Balami J. S. Esiri M. M. Chen L. K. Buchan A. M. (2010). Ischemic stroke in the elderly: An overview of evidence. *Nat. Rev. Neurol.* 6 256–265. 10.1038/nrneurol.2010.36 20368741

[B23] Chen X. Feng W. Ou R. Liu J. Yang J. Fu J. (2021). Evidence for peripheral immune activation in Parkinson’s disease. *Front. Aging Neurosci.* 13:617370. 10.3389/fnagi.2021.617370 33994989 PMC8119625

[B24] Chen X. Firulyova M. Manis M. Herz J. Smirnov I. Aladyeva E. (2023). Microglia-mediated T cell infiltration drives neurodegeneration in tauopathy. *Nature* 615 668–677. 10.1038/s41586-023-05788-0 36890231 PMC10258627

[B25] Coque E. Salsac C. Espinosa-Carrasco G. Varga B. Degauque N. Cadoux M. (2019). Cytotoxic CD8(+) T lymphocytes expressing ALS-causing SOD1 mutant selectively trigger death of spinal motoneurons. *Proc. Natl. Acad. Sci. U.S.A.* 116 2312–2317. 10.1073/pnas.1815961116 30674678 PMC6369778

[B26] Cramer S. C. Riley J. D. (2008). Neuroplasticity and brain repair after stroke. *Curr. Opin. Neurol.* 21 76–82. 10.1097/WCO.0b013e3282f36cb6 18180655

[B27] Daglas M. Draxler D. F. Ho H. McCutcheon F. Galle A. Au A. E. (2019). Activated CD8(+) T cells cause long-term neurological impairment after traumatic brain injury in mice. *Cell Rep.* 29 1178.e6–1191.e6. 10.1016/j.celrep.2019.09.046 31665632

[B28] Dugger B. N. Dickson D. W. (2017). Pathology of neurodegenerative diseases. *Cold Spring Harbor Perspect. Biol.* 9:a028035. 10.1101/cshperspect.a028035 28062563 PMC5495060

[B29] Ellwardt E. Walsh J. T. Kipnis J. Zipp F. (2016). Understanding the role of T cells in CNS homeostasis. *Trends Immunol.* 37 154–165. 10.1016/j.it.2015.12.008 26775912

[B30] Evans F. L. Dittmer M. de la Fuente A. G. Fitzgerald D. C. (2019). Protective and regenerative roles of T cells in central nervous system disorders. *Front. Immunol.* 10:2171. 10.3389/fimmu.2019.02171 31572381 PMC6751344

[B31] Fan L. Zhang C. J. Zhu L. Chen J. Zhang Z. Liu P. (2020). FASL-PDPK1 pathway promotes the cytotoxicity of CD8(+) T cells during ischemic stroke. *Trans. Stroke Res.* 11 747–761. 10.1007/s12975-019-00749-0 32036560

[B32] Fernando N. Gopalakrishnan J. Behensky A. Reich L. Liu C. Bass V. (2023). Single-cell multiomic analysis reveals the involvement of Type I interferon-responsive CD8+ T cells in amyloid beta-associated memory loss. *bioRxiv*. [Preprint]. 10.1101/2023.03.18.533293 37090642 PMC10120715

[B33] Fiala M. Chattopadhay M. La Cava A. Tse E. Liu G. Lourenco E. (2010). IL-17A is increased in the serum and in spinal cord CD8 and mast cells of ALS patients. *J. Neuroinflammation* 7:76. 10.1186/1742-2094-7-76 21062492 PMC2992053

[B34] Finlay D. K. Rosenzweig E. Sinclair L. V. Feijoo-Carnero C. Hukelmann J. L. Rolf J. (2012). PDK1 regulation of mTOR and hypoxia-inducible factor 1 integrate metabolism and migration of CD8+ T cells. *J. Exp. Med.* 209 2441–2453. 10.1084/jem.20112607 23183047 PMC3526360

[B35] Fu L. Zhou X. Jiao Q. Chen X. (2023). The functions of TRIM56 in antiviral innate immunity and tumorigenesis. *Int. J. Mol. Sci.* 24:5046. 10.3390/ijms24055046 36902478 PMC10003129

[B36] Galiano-Landeira J. Torra A. Vila M. Bové J. (2020). CD8 T cell nigral infiltration precedes synucleinopathy in early stages of Parkinson’s disease. *Brain* 143 3717–3733. 10.1093/brain/awaa269 33118032

[B37] Gate D. Saligrama N. Leventhal O. Yang A. C. Unger M. S. Middeldorp J. (2020). Clonally expanded CD8 T cells patrol the cerebrospinal fluid in Alzheimer’s disease. *Nature* 577 399–404. 10.1038/s41586-019-1895-7 31915375 PMC7445078

[B38] Gelderblom M. Leypoldt F. Steinbach K. Behrens D. Choe C. U. Siler D. A. (2009). Temporal and spatial dynamics of cerebral immune cell accumulation in stroke. *Stroke* 40 1849–1857. 10.1161/STROKEAHA.108.534503 19265055

[B39] Gerlach C. van Heijst J. W. Swart E. Sie D. Armstrong N. Kerkhoven R. M. (2010). One naive T cell, multiple fates in CD8+ T cell differentiation. *J. Exp. Med.* 207 1235–1246. 10.1084/jem.20091175 20479114 PMC2882844

[B40] Golstein P. Griffiths G. M. (2018). An early history of T cell-mediated cytotoxicity. *Nat. Rev. Immunol.* 18 527–535. 10.1038/s41577-018-0009-3 29662120

[B41] Halliday G. (2020). Neglected cytotoxic T cell invasion of the brain: How specific for Parkinson’s disease? *Brain* 143 3518–3521. 10.1093/brain/awaa390 33439981

[B42] Hazeldine J. Lord J. M. Belli A. (2015). Traumatic brain injury and peripheral immune suppression: Primer and prospectus. *Front. Neurol.* 6:235. 10.3389/fneur.2015.00235 26594196 PMC4633482

[B43] He Y. Peng K. Li R. Zhang Z. Pan L. Zhang T. (2022). Changes of T lymphocyte subpopulations and their roles in predicting the risk of Parkinson’s disease. *J. Neurol.* 269 5368–5381. 10.1007/s00415-022-11190-z 35608657 PMC9467943

[B44] Heming M. Börsch A. L. Wiendl H. Meyer zu Hörste G. M. (2022). High-dimensional investigation of the cerebrospinal fluid to explore and monitor CNS immune responses. *Genome Med.* 14:94. 10.1186/s13073-022-01097-9 35978442 PMC9385102

[B45] Heneka M. T. (2020). An immune-cell signature marks the brain in Alzheimer’s disease. *Nature* 577 322–323. 10.1038/d41586-019-03892-8 31937952

[B46] Hisanaga K. Asagi M. Itoyama Y. Iwasaki Y. (2001). Increase in peripheral CD4 bright+ CD8 dull+ T cells in Parkinson disease. *Arch. Neurol.* 58 1580–1583. 10.1001/archneur.58.10.1580 11594915

[B47] Hobson B. D. Sulzer D. (2022). Neuronal presentation of antigen and its possible role in Parkinson’s disease. *J. Parkinson’s Dis.* 12 S137–S147. 10.3233/JPD-223153 35253783 PMC9440948

[B48] Hobson R. Levy S. H. S. Flaherty D. Xiao H. Ciener B. Reddy H. (2023). Clonal CD8 T cells in the leptomeninges are locally controlled and influence microglia in human neurodegeneration. *bioRxiv.* [Preprint]. 10.1101/2023.07.13.548931 37503131 PMC10369982

[B49] Houser M. C. Caudle W. M. Chang J. Kannarkat G. T. Yang Y. Kelly S. D. (2021). Experimental colitis promotes sustained, sex-dependent, T-cell-associated neuroinflammation and parkinsonian neuropathology. *Acta Neuropathol. Commun.* 9 139. 10.1186/s40478-021-01240-4 34412704 PMC8375080

[B50] Hua R. Mao S. S. Zhang Y. M. Chen F. X. Zhou Z. H. Liu J. Q. (2012). Effects of pituitary adenylate cyclase activating polypeptide on CD4(+)/CD8(+) T cell levels after traumatic brain injury in a rat model. *World J. Emerg. Med.* 3 294–298. 10.5847/wjem.j.issn.1920-8642.2012.04.010 25215080 PMC4129813

[B51] Iadecola C. Anrather J. (2011). The immunology of stroke: From mechanisms to translation. *Nat. Med.* 17 796–808. 10.1038/nm.2399 21738161 PMC3137275

[B52] Iadecola C. Buckwalter M. S. Anrather J. (2020). Immune responses to stroke: Mechanisms, modulation, and therapeutic potential. *J. Clin. Invest.* 130 2777–2788. 10.1172/JCI135530 32391806 PMC7260029

[B53] Ip C. W. Beck S. K. Volkmann J. (2015). Lymphocytes reduce nigrostriatal deficits in the 6-hydroxydopamine mouse model of Parkinson’s disease. *J. Neural Trans.* 122 1633–1643. 10.1007/s00702-015-1444-y 26290125

[B54] Itagaki S. McGeer P. L. Akiyama H. (1988). Presence of T-cytotoxic suppressor and leucocyte common antigen positive cells in Alzheimer’s disease brain tissue. *Neurosci. Lett.* 91 259–264. 10.1016/0304-3940(88)90690-8 2972943

[B55] Jameson S. C. Masopust D. (2009). Diversity in T cell memory: An embarrassment of riches. *Immunity* 31 859–871. 10.1016/j.immuni.2009.11.007 20064446 PMC2957815

[B56] Jayaraj R. L. Azimullah S. Beiram R. Jalal F. Y. Rosenberg G. A. (2019). Neuroinflammation: Friend and foe for ischemic stroke. *J. Neuroinflammation* 16:142. 10.1186/s12974-019-1516-2 31291966 PMC6617684

[B57] Jiang S. S. Wang Y. L. Xu Q. H. Gu L. Y. Kang R. Q. Yang W. Y. (2023). Cytokine and chemokine map of peripheral specific immune cell subsets in Parkinson’s disease. *Parkinson’s Dis.* 9 117. 10.1038/s41531-023-00559-0 37491350 PMC10368737

[B58] Jiang X. Xu J. Liu M. Xing H. Wang Z. Huang L. (2019). Adoptive CD8(+) T cell therapy against cancer:challenges and opportunities. *Cancer Lett.* 462 23–32. 10.1016/j.canlet.2019.07.017 31356845

[B59] Jovic D. Liang X. Zeng H. Lin L. Xu F. Luo Y. (2022). Single-cell RNA sequencing technologies and applications: A brief overview. *Clin. Transl. Med.* 12:e694. 10.1002/ctm2.694 35352511 PMC8964935

[B60] Kaech S. M. Cui W. (2012). Transcriptional control of effector and memory CD8+ T cell differentiation. *Nat. Revi. Immunol.* 12 749–761. 10.1038/nri3307 23080391 PMC4137483

[B61] Kaech S. M. Wherry E. J. (2007). Heterogeneity and cell-fate decisions in effector and memory CD8+ T cell differentiation during viral infection. *Immunity* 27 393–405. 10.1016/j.immuni.2007.08.007 17892848 PMC3431921

[B62] Kaur K. Chen P. C. Ko M. W. Mei A. Chovatiya N. Huerta-Yepez S. (2022). The potential role of cytotoxic immune effectors in induction, progression and pathogenesis of amyotrophic lateral sclerosis (ALS). *Cells* 11:3431. 10.3390/cells11213431 36359827 PMC9656116

[B63] Kim H. R. Hwang K. A. Park S. H. Kang I. (2008). IL-7 and IL-15: Biology and roles in T-cell immunity in health and disease. *Crit. Rev. Immunol.* 28 325–339. 10.1615/critrevimmunol.v28.i4.40 19166383

[B64] Kouli A. Jensen M. Papastavrou V. Scott K. M. Kolenda C. Parker C. (2021). T lymphocyte senescence is attenuated in Parkinson’s disease. *J. Neuroinflammation* 18:228. 10.1186/s12974-021-02287-9 34645462 PMC8513368

[B65] Kumar B. V. Connors T. J. Farber D. L. (2018). Human T cell development, localization, and function throughout life. *Immunity* 48 202–213. 10.1016/j.immuni.2018.01.007 29466753 PMC5826622

[B66] Kumari R. Gensel J. C. (2023). Microglia as drivers of neurodegeneration: The role of innate-adaptive immune signaling. *Neuron* 111 597–598. 10.1016/j.neuron.2023.02.005 36863316

[B67] Kureshi S. A. Hofman F. M. Schneider J. H. Chin L. S. Apuzzo M. L. Hinton D. R. (1994). Cytokine expression in radiation-induced delayed cerebral injury. *Neurosurgery* 35 822–829. 10.1227/00006123-199411000-00004 7838329

[B68] Larbi A. Pawelec G. Witkowski J. M. Schipper H. M. Derhovanessian E. Goldeck D. (2009). Dramatic shifts in circulating CD4 but not CD8 T cell subsets in mild Alzheimer’s disease. *J. Alzheimer’s Dis.* 17 91–103. 10.3233/JAD-2009-1015 19494434

[B69] Laurent C. Dorothée G. Hunot S. Martin E. Monnet Y. Duchamp M. (2017). Hippocampal T cell infiltration promotes neuroinflammation and cognitive decline in a mouse model of tauopathy. *Brain* 140 184–200. 10.1093/brain/aww270 27818384 PMC5382942

[B70] Leavy A. Jimenez Mateos E. M. (2020). Perinatal brain injury and inflammation: Lessons from experimental murine models. *Cells* 9:2640. 10.3390/cells9122640 33302543 PMC7764185

[B71] Lee G. A. Lin T. N. Chen C. Y. Mau S. Y. Huang W. Z. Kao Y. C. (2018). Interleukin 15 blockade protects the brain from cerebral ischemia-reperfusion injury. *Brain Behav. Immun.* 73 562–570. 10.1016/j.bbi.2018.06.021 29959050

[B72] Lei J. Xie L. Zhao H. Gard C. Clemens J. L. McLane M. W. (2018). Maternal CD8(+) T-cell depletion alleviates intrauterine inflammation-induced perinatal brain injury. *Ame. J. Reproduct. Immunol.* 79:e12798. 10.1111/aji.12798 29205631 PMC5908745

[B73] Li M. Li Z. Yao Y. Jin W. N. Wood K. Liu Q. (2017). Astrocyte-derived interleukin-15 exacerbates ischemic brain injury via propagation of cellular immunity. *Proc. Natl. Acad. Scie. U.S.A.* 114 E396–E405. 10.1073/pnas.1612930114 27994144 PMC5255606

[B74] Li X. Chen G. (2023). CNS-peripheral immune interactions in hemorrhagic stroke. *J. Cereb. Blood Flow Metab.* 43 185–197. 10.1177/0271678X221145089 36476130 PMC9903219

[B75] Ling C. Sandor M. Suresh M. Fabry Z. (2006). Traumatic injury and the presence of antigen differentially contribute to T-cell recruitment in the CNS. *J. Neurosci.* 26 731–741. 10.1523/JNEUROSCI.3502-05.2006 16421293 PMC6675378

[B76] Lueg G. Gross C. C. Lohmann H. Johnen A. Kemmling A. Deppe M. (2015). Clinical relevance of specific T-cell activation in the blood and cerebrospinal fluid of patients with mild Alzheimer’s disease. *Neurobiol. Aging* 36 81–89. 10.1016/j.neurobiolaging.2014.08.008 25277040

[B77] Marsh S. E. Abud E. M. Lakatos A. Karimzadeh A. Yeung S. T. Davtyan H. (2016). The adaptive immune system restrains Alzheimer’s disease pathogenesis by modulating microglial function. *Proc. Natl. Acad. Sci. U.S.A.* 113 E1316–E1325. 10.1073/pnas.1525466113 26884167 PMC4780638

[B78] McKee A. C. Daneshvar D. H. (2015). The neuropathology of traumatic brain injury. *Handb. Clin. Neurol.* 127 45–66. 10.1016/B978-0-444-52892-6.00004-0 25702209 PMC4694720

[B79] McKinney E. F. Cuthbertson I. Harris K. M. Smilek D. E. Connor C. Manferrari G. (2021). A CD8(+) NK cell transcriptomic signature associated with clinical outcome in relapsing remitting multiple sclerosis. *Nat. Commun.* 12:635. 10.1038/s41467-020-20594-2 33504809 PMC7840761

[B80] McManus R. M. Heneka M. T. (2020). T cells in Alzheimer’s disease: Space invaders. *Lancet Neurol.* 19 285–287. 10.1016/S1474-4422(20)30076-4 32199089

[B81] McQuillan K. Lynch M. A. Mills K. H. G. (2010). Activation of mixed glia by Abeta-specific Th1 and Th17 cells and its regulation by Th2 cells. *Brain Behav. Immun.* 24 598–607. 10.1016/j.bbi.2010.01.003 20060887

[B82] Merlini M. Kirabali T. Kulic L. Nitsch R. M. Ferretti M. T. (2018). Extravascular CD3+ T cells in brains of Alzheimer disease patients correlate with tau but not with amyloid pathology: An immunohistochemical study. *Neuro Degener. Dis.* 18 49–56. 10.1159/000486200 29402847

[B83] Michael J. Zirknitzer J. Unger M. S. Poupardin R. Rieß T. Paiement N. (2021). The leukotriene receptor antagonist montelukast attenuates neuroinflammation and affects cognition in transgenic 5xFAD mice. *Int. J. Mol. Sci.* 22:2782. 10.3390/ijms22052782 33803482 PMC7967180

[B84] Mittrücker H. W. Visekruna A. Huber M. (2014). Heterogeneity in the differentiation and function of CD8(+) T cells. *Arch. Immunol. Ther. Exp.* 62 449–458. 10.1007/s00005-014-0293-y 24879097

[B85] Montaño A. Hanley D. F. Hemphill J. C. III (2021). Hemorrhagic stroke. *Handb. Clin. Neurol.* 176 229–248. 10.1016/B978-0-444-64034-5.00019-5 33272397

[B86] Morganti-Kossmann M. C. Semple B. D. Hellewell S. C. Bye N. Ziebell J. M. (2019). The complexity of neuroinflammation consequent to traumatic brain injury: From research evidence to potential treatments. *Acta Neuropathol.* 137 731–755. 10.1007/s00401-018-1944-6 30535946

[B87] Mracsko E. Javidi E. Na S. Y. Kahn A. Liesz A. Veltkamp R. (2014a). Leukocyte invasion of the brain after experimental intracerebral hemorrhage in mice. *Stroke* 45 2107–2114. 10.1161/STROKEAHA.114.005801 24916913

[B88] Mracsko E. Liesz A. Stojanovic A. Lou W. P. Osswald M. Zhou W. (2014b). Antigen dependently activated cluster of differentiation 8-positive T cells cause perforin-mediated neurotoxicity in experimental stroke. *J. Neurosci.* 34 16784–16795. 10.1523/JNEUROSCI.1867-14.2014 25505331 PMC6608504

[B89] Nardo G. Trolese M. C. Verderio M. Mariani A. de Paola M. Riva N. (2018). Counteracting roles of MHCI and CD8(+) T cells in the peripheral and central nervous system of ALS SOD1(G93A) mice. *Mol. Neurodegener.* 13:42. 10.1186/s13024-018-0271-7 30092791 PMC6085701

[B90] Nasa P. (2022). Outcome of hemorrhagic stroke: Host immune response can be a prediction tool! *Indian J. Crit. Care Med.* 26 2–4. 10.5005/jp-journals-10071-24095 35110832 PMC8783247

[B91] Novak C. M. Ozen M. Burd I. (2018). Perinatal brain injury: Mechanisms, prevention, and outcomes. *Clin. Perinatol.* 45 357–375. 10.1016/j.clp.2018.01.015 29747893

[B92] Ohashi S. N. DeLong J. H. Kozberg M. G. Mazur-Hart D. J. van Veluw S. J. Alkayed N. J. (2023). Role of inflammatory processes in hemorrhagic stroke. *Stroke* 54 605–619. 10.1161/STROKEAHA.122.037155 36601948

[B93] Pan J. Konstas A. A. Bateman B. Ortolano G. A. Pile-Spellman J. (2007). Reperfusion injury following cerebral ischemia: Pathophysiology, MR imaging, and potential therapies. *Neuroradiology* 49 93–102. 10.1007/s00234-006-0183-z 17177065 PMC1786189

[B94] Philip M. Schietinger A. (2022). CD8(+) T cell differentiation and dysfunction in cancer. *Nat. Rev. Immunol.* 22 209–223. 10.1038/s41577-021-00574-3 34253904 PMC9792152

[B95] Pirttilä T. Mattinen S. Frey H. (1992). The decrease of CD8-positive lymphocytes in Alzheimer’s disease. *J. Neurol. Sci.* 107 160–165. 10.1016/0022-510x(92)90284-r 1564514

[B96] Popovich P. G. van Rooijen N. Hickey W. F. Preidis G. McGaughy V. (2003). Hematogenous macrophages express CD8 and distribute to regions of lesion cavitation after spinal cord injury. *Exp. Neurol.* 182 275–287. 10.1016/S0014-4886(03)00120-1 12895439

[B97] Rauschenberger L. Behnke J. Grotemeyer A. Knorr S. Volkmann J. Ip C. W. (2022). Age-dependent neurodegeneration and neuroinflammation in a genetic A30P/A53T double-mutated alpha-synuclein mouse model of Parkinson’s disease. *Neurobiol. Dis.* 171:105798. 10.1016/j.nbd.2022.105798 35750147

[B98] Reina-Campos M. Scharping N. E. Goldrath A. W. (2021). CD8(+) T cell metabolism in infection and cancer. *Nat. Rev. Immunol.* 21 718–738. 10.1038/s41577-021-00537-8 33981085 PMC8806153

[B99] Reiss J. D. Peterson L. S. Nesamoney S. N. Chang A. L. Pasca A. M. Marić I. (2022). Perinatal infection, inflammation, preterm birth, and brain injury: A review with proposals for future investigations. *Exp. Neurol.* 351:113988. 10.1016/j.expneurol.2022.113988 35081400

[B100] Ribeiro F. M. (2023). Understanding brain diseases: From receptor dysregulation to neurodegeneration, neuroinflammation and memory impairment. *Curr. Neuropharmacol.* 21 162–163. 10.2174/1570159X2102221212143233 36859816 PMC10190147

[B101] Ribeiro M. Brigas H. C. Temido-Ferreira M. Pousinha P. A. Regen T. Santa C. (2019). Meningeal gammadelta T cell-derived IL-17 controls synaptic plasticity and short-term memory. *Sci. Immunol.* 4:eaay5199. 10.1126/sciimmunol.aay5199 31604844 PMC6894940

[B102] Ritzel R. M. Crapser J. Patel A. R. Verma R. Grenier J. M. Chauhan A. (2016). Age-associated resident memory CD8 T cells in the central nervous system are primed to potentiate inflammation after ischemic brain injury. *J. Immunol.* 196 3318–3330. 10.4049/jimmunol.1502021 26962232 PMC4868658

[B103] Ritzel R. M. Doran S. J. Barrett J. P. Henry R. J. Ma E. L. Faden A. I. (2018). Chronic alterations in systemic immune function after traumatic brain injury. *J. Neurotrauma* 35 1419–1436. 10.1089/neu.2017.5399 29421977 PMC5998829

[B104] Salvador A. F. M. Kipnis J. (2022). Immune response after central nervous system injury. *Semin. Immunol.* 59:101629. 10.1016/j.smim.2022.101629 35753867

[B105] Schindowski K. Eckert A. Peters J. Gorriz C. Schramm U. Weinandi T. (2007). Increased T-cell reactivity and elevated levels of CD8+ memory T-cells in Alzheimer’s disease-patients and T-cell hyporeactivity in an Alzheimer’s disease-mouse model: Implications for immunotherapy. *NeuroMol. Med.* 9 340–354. 10.1007/s12017-007-8015-9 17963048

[B106] Selvaraj U. M. Ujas T. A. Kong X. Kumar A. Plautz E. J. Zhang S. (2021). Delayed diapedesis of CD8 T cells contributes to long-term pathology after ischemic stroke in male mice. *Brain Behav. Immun.* 95 502–513. 10.1016/j.bbi.2021.05.001 33964435 PMC8221572

[B107] Shalit F. Sredni B. Brodie C. Kott E. Huberman M. (1995). T lymphocyte subpopulations and activation markers correlate with severity of Alzheimer’s disease. *Clin. Immunol. Immunopathol.* 75 246–250. 10.1006/clin.1995.1078 7768042

[B108] Sheridan B. S. Lefrançois L. (2011). Regional and mucosal memory T cells. *Nat. Immunol.* 12 485–491. 10.1038/ni.2029 21739671 PMC3224372

[B109] Shi N. Kawano Y. Tateishi T. Kikuchi H. Osoegawa M. Ohyagi Y. (2007). Increased IL-13-producing T cells in ALS: Positive correlations with disease severity and progression rate. *J. Neuroimmunol.* 182 232–235. 10.1016/j.jneuroim.2006.10.001 17097743

[B110] Shi Z. Yu P. Lin W. J. Chen S. Hu X. Chen S. (2023). Microglia drive transient insult-induced brain injury by chemotactic recruitment of CD8(+) T lymphocytes. *Neuron* 111 696.e9–710.e9. 10.1016/j.neuron.2022.12.009 36603584

[B111] Sinha S. Boyden A. W. Itani F. R. Crawford M. P. Karandikar N. J. (2015). CD8(+) T-cells as immune regulators of multiple sclerosis. *Front. Immunol.* 6:619. 10.3389/fimmu.2015.00619 26697014 PMC4674574

[B112] Speciale L. Calabrese E. Saresella M. Tinelli C. Mariani C. Sanvito L. (2007). Lymphocyte subset patterns and cytokine production in Alzheimer’s disease patients. *Neurobiol. Aging* 28 1163–1169. 10.1016/j.neurobiolaging.2006.05.020 16814429

[B113] Speiser D. E. Ho P. C. Verdeil G. (2016). Regulatory circuits of T cell function in cancer. *Nat. Rev. Immunol.* 16 599–611. 10.1038/nri.2016.80 27526640

[B114] Sribnick E. A. Popovich P. G. Hall M. W. (2022). Central nervous system injury-induced immune suppression. *Neurosurg. Focus* 52:E10. 10.3171/2021.11.FOCUS21586 35104790 PMC8931741

[B115] St Paul M. Ohashi P. S. (2020). The roles of CD8(+) T cell subsets in antitumor immunity. *Trends Cell Biol.* 30 695–704. 10.1016/j.tcb.2020.06.003 32624246

[B116] Stampanoni Bassi M. Iezzi E. Centonze D. (2022). Multiple sclerosis: Inflammation, autoimmunity and plasticity. *Handb. Clin. Neurol.* 184 457–470. 10.1016/B978-0-12-819410-2.00024-2 35034754

[B117] Su W. Saravia J. Risch I. Rankin S. Guy C. Chapman N. M. (2023). CXCR6 orchestrates brain CD8(+) T cell residency and limits mouse Alzheimer’s disease pathology. *Nat. Immunol.* 24 1735–1747. 10.1038/s41590-023-01604-z 37679549 PMC11102766

[B118] Sulzer D. Alcalay R. N. Garretti F. Cote L. Kanter E. Agin-Liebes J. (2017). T cells from patients with Parkinson’s disease recognize alpha-synuclein peptides. *Nature* 546 656–661. 10.1038/nature22815 28636593 PMC5626019

[B119] Sun L. Su Y. Jiao A. Wang X. Zhang B. (2023). T cells in health and disease. *Signal Trans. Target. Ther.* 8 235. 10.1038/s41392-023-01471-y 37332039 PMC10277291

[B120] Surh C. D. Sprent J. (2008). Homeostasis of naive and memory T cells. *Immunity* 29 848–862. 10.1016/j.immuni.2008.11.002 19100699

[B121] Tabilas C. Smith N. L. Rudd B. D. (2023). Shaping immunity for life: Layered development of CD8(+) T cells. *Immunol. Rev.* 315 108–125. 10.1111/imr.13185 36653953 PMC10205662

[B122] Taylor C. A. Bell J. M. Breiding M. J. Xu L. (2017). Traumatic brain injury-related emergency department visits, hospitalizations, and deaths – United States, 2007 and 2013. Morbidity and mortality weekly report. *Surveill. Summ.* 66 1–16. 10.15585/mmwr.ss6609a1 28301451 PMC5829835

[B123] Thakur P. Breger L. S. Lundblad M. Wan O. W. Mattsson B. Luk K. C. (2017). Modeling Parkinson’s disease pathology by combination of fibril seeds and alpha-synuclein overexpression in the rat brain. *Proc. Natl. Acad. Sci. U.S.A.* 114 E8284–E8293. 10.1073/pnas.1710442114 28900002 PMC5625925

[B124] Unger M. S. Li E. Scharnagl L. Poupardin R. Altendorfer B. Mrowetz H. (2020). CD8(+) T-cells infiltrate Alzheimer’s disease brains and regulate neuronal- and synapse-related gene expression in APP-PS1 transgenic mice. *Brain Behav. Immun.* 89 67–86. 10.1016/j.bbi.2020.05.070 32479993

[B125] Vlisides P. Mashour G. A. (2016). Perioperative stroke. *Can. J. Anesthesia* 63 193–204. 10.1007/s12630-015-0494-9 26391795 PMC4720532

[B126] Wang B. Y. Ye Y. Y. Qian C. Zhang H. B. Mao H. X. Yao L. P. (2021). Stress increases MHC-I expression in dopaminergic neurons and induces autoimmune activation in Parkinson’s disease. *Neural Regenerati. Res.* 16 2521–2527. 10.4103/1673-5374.313057 33907043 PMC8374590

[B127] Wang P. Yao L. Luo M. Zhou W. Jin X. Xu Z. (2021). Single-cell transcriptome and TCR profiling reveal activated and expanded T cell populations in Parkinson’s disease. *Cell Discov.* 7:52. 10.1038/s41421-021-00280-3 34282123 PMC8289849

[B128] Wang Y. R. Cui W. Q. Wu H. Y. Xu X. D. Xu X. Q. (2023). The role of T cells in acute ischemic stroke. *Brain Res. Bull.* 196 20–33. 10.1016/j.brainresbull.2023.03.005 36906042

[B129] Wareham L. K. Liddelow S. A. Temple S. Benowitz L. I. Di Polo A. Wellington C. (2022). Solving neurodegeneration: Common mechanisms and strategies for new treatments. *Mol. Neurodegener.* 17:23. 10.1186/s13024-022-00524-0 35313950 PMC8935795

[B130] Westman G. Lidehall A. K. Magnusson P. Ingelsson M. Kilander L. Lannfelt L. (2013). Decreased proportion of cytomegalovirus specific CD8 T-cells but no signs of general immunosenescence in Alzheimer’s disease. *PLoS One* 8:e77921. 10.1371/journal.pone.0077921 24155977 PMC3796487

[B131] Williams G. P. Muskat K. Frazier A. Xu Y. Mateus J. Grifoni A. (2023). Unaltered T cell responses to common antigens in individuals with Parkinson’s disease. *J. Neurol. Sci.* 444:120510. 10.1016/j.jns.2022.120510 36495691 PMC9950758

[B132] Williams-Gray C. H. Wijeyekoon R. S. Scott K. M. Hayat S. Barker R. A. Jones J. L. (2018). Abnormalities of age-related T cell senescence in Parkinson’s disease. *J. Neuroinflammation* 15:166. 10.1186/s12974-018-1206-5 29807534 PMC5972443

[B133] Wilson D. M. III Cookson M. R. Van Den Bosch L. Zetterberg H. Holtzman D. M. Dewachter I. (2023). Hallmarks of neurodegenerative diseases. *Cell* 186 693–714. 10.1016/j.cell.2022.12.032 36803602

[B134] Wu L. Ji N. N. Wang H. Hua J. Y. Sun G. L. Chen P. P. (2021). Domino effect of interleukin-15 and CD8 T-cell-mediated neuronal apoptosis in experimental traumatic brain injury. *J. Neurotrauma* 38 1450–1463. 10.1089/neu.2017.5607 30430911

[B135] Xie L. Li W. Hersh J. Liu R. Yang S. H. (2019). Experimental ischemic stroke induces long-term T cell activation in the brain. *J. Cereb. Blood Flow Metab.* 39 2268–2276. 10.1177/0271678X18792372 30092705 PMC6827125

[B136] Xu L. Ye X. Wang Q. Xu B. Zhong J. Chen Y. Y. (2021). T-cell infiltration, contribution and regulation in the central nervous system post-traumatic injury. *Cell Prolif.* 54:e13092. 10.1111/cpr.13092 34189783 PMC8349661

[B137] Xue M. Del Bigio M. R. (2003). Comparison of brain cell death and inflammatory reaction in three models of intracerebral hemorrhage in adult rats. *J. Stroke Cerebrovas. Dis.* 12 152–159. 10.1016/S1052-3057(03)00036-3 17903920

[B138] Yang Y. Ye Y. Chen C. Kong C. Su X. Zhang X. (2019). Acute traumatic brain injury induces CD4+ and CD8+ T cell functional impairment by upregulating the expression of PD-1 via the activated sympathetic nervous system. *Neuroimmunomodulation* 26 43–57. 10.1159/000495465 30695785

[B139] Yazdani S. Seitz C. Cui C. Lovik A. Pan L. Piehl F. (2022). T cell responses at diagnosis of amyotrophic lateral sclerosis predict disease progression. *Nat. Commun.* 13:6733. 10.1038/s41467-022-34526-9 36347843 PMC9643478

[B140] Ye X. Chen J. Pan J. Wu Q. Wang Y. Lu M. (2023). Interleukin-17 promotes the infiltration of CD8+ T cells into the brain in a mouse model for Alzheimer’s disease. *Immunol. Invest.* 52 135–153. 10.1080/08820139.2022.2136525 36394561

[B141] Yoritsune E. Furuse M. Kuwabara H. Miyata T. Nonoguchi N. Kawabata S. (2014). Inflammation as well as angiogenesis may participate in the pathophysiology of brain radiation necrosis. *J. Radiat. Res.* 55 803–811. 10.1093/jrr/rru017 24676944 PMC4100008

[B142] Zhang F. Niu M. Guo K. Ma Y. Fu Q. Liu Y. (2022). The immunometabolite S-2-hydroxyglutarate exacerbates perioperative ischemic brain injury and cognitive dysfunction by enhancing CD8(+) T lymphocyte-mediated neurotoxicity. *J. Neuroinflammation* 19:176. 10.1186/s12974-022-02537-4 35799259 PMC9264651

[B143] Zhang J. Ke K. F. Liu Z. Qiu Y. H. Peng Y. P. (2013). Th17 cell-mediated neuroinflammation is involved in neurodegeneration of abeta1-42-induced Alzheimer’s disease model rats. *PLoS One* 8:e75786. 10.1371/journal.pone.0075786 24124514 PMC3790825

[B144] Zhang T. Warden A. R. Li Y. Ding X. (2020). Progress and applications of mass cytometry in sketching immune landscapes. *Clin. Transl. Med.* 10:e206. 10.1002/ctm2.206 33135337 PMC7556381

[B145] Zhang Z. Artelt M. Burnet M. Trautmann K. Schluesener H. J. (2006). Early infiltration of CD8+ macrophages/microglia to lesions of rat traumatic brain injury. *Neuroscience* 141 637–644. 10.1016/j.neuroscience.2006.04.027 16725271

[B146] Zhang Z. Lv M. Zhou X. Cui Y. (2022). Roles of peripheral immune cells in the recovery of neurological function after ischemic stroke. *Front. Cell. Neurosci.* 16:1013905. 10.3389/fncel.2022.1013905 36339825 PMC9634819

[B147] Zhao H. Xie L. Clemens J. L. Zong L. McLane M. W. Arif H. (2020). Mouse bone marrow-derived mesenchymal stem cells alleviate perinatal brain injury via a CD8(+) T cell mechanism in a model of intrauterine inflammation. *Reproduct. Sci.* 27 1465–1476. 10.1007/s43032-020-00157-y 31997258

[B148] Zhou Y. X. Wang X. Tang D. Li Y. Jiao Y. F. Gan Y. (2019). IL-2mAb reduces demyelination after focal cerebral ischemia by suppressing CD8(+) T cells. *CNS Neurosci. Ther.* 25 532–543. 10.1111/cns.13084 30444079 PMC6488908

